# Enhancing the Photocurrent of Top-Cell by Ellipsoidal Silver Nanoparticles: Towards Current-Matched GaInP/GaInAs/Ge Triple-Junction Solar Cells

**DOI:** 10.3390/nano6060098

**Published:** 2016-05-25

**Authors:** Yiming Bai, Lingling Yan, Jun Wang, Lin Su, Zhigang Yin, Nuofu Chen, Yuanyuan Liu

**Affiliations:** 1 State Key Laboratory of Alternate Electrical Power System with Renewable Energy Sources, North China Electric Power University, Beijing 102206, China; dgunwung@163.com (L.Y.); 18229799348@163.com (L.S.); 2 Institute of Information Photonics and Optical Communications, Beijing University of Posts and Telecommunications, Beijing 100876, China; wangjun@semi.ac.cn; 3 Key Laboratory of Semiconductor Materials Science, Institute of Semiconductors, Chinese Academy of Sciences, P.O. Box 912, Beijing 100083, China; yzhg@semi.ac.cn (Z.Y.); liuyy@semi.ac.cn (Y.L.)

**Keywords:** GaInP/GaInAs/Ge triple-junction solar cells, Ag ellipsoidal nanoparticles, thermal treatment parameters, current-match

## Abstract

A way to increase the photocurrent of top-cell is crucial for current-matched and highly-efficient GaInP/GaInAs/Ge triple-junction solar cells. Herein, we demonstrate that ellipsoidal silver nanoparticles (Ag NPs) with better extinction performance and lower fabrication temperature can enhance the light harvest of GaInP/GaInAs/Ge solar cells compared with that of spherical Ag NPs. In this method, appropriate thermal treatment parameters for Ag NPs without inducing the dopant diffusion of the tunnel-junction plays a decisive role. Our experimental and theoretical results confirm the ellipsoidal Ag NPs annealed at 350 °C show a better extinction performance than the spherical Ag NPs annealed at 400 °C. The photovoltaic conversion efficiency of the device with ellipsoidal Ag NPs reaches 31.02%, with a nearly 5% relative improvement in comparison with the device without Ag NPs (29.54%). This function of plasmonic NPs has the potential to solve the conflict of sufficient light absorption and efficient carrier collection in GaInP top-cell devices.

## 1. Introduction

Currently, GaInP/GaInAs/Ge triple-junction solar cells (TJSCs) for space and terrestrial concentrator applications have attracted increasing attention for their very high conversion efficiencies [1,2,3] and dramatic reduction in cost [4]. Such TJSCs with different subcell bandgaps divide the broad solar spectrum into three narrower sections, each of which can be converted to electricity more efficiently [5,6], while the 50% theoretical efficiency have not been obtained as expected due to the current mismatch among the subcells [7,8]. There are two aspects of this problem that have to be addressed. The first involves the Ge subcell, which absorbs approximately two times that photons than that needed for current matching with the GaInP and GaAs subcells [6]. The second problem relates to the lowest photocurrent of GaInP top-cells, which limits the efficiency of TJSCs greatly [9]. To the former, some reports suggest that the Ge bottom-cell would be replaced by a material with a bandgap of 1.0 eV, such as GaInNAs [10], but it is confined by the requirements of lattice matching with the other junctions and a higher epitaxy technique. Hence, improving the photocurrent of GaInP top-cell to approach other subcells is an effective way for enhancing the conversion efficiency of TJSCs.

For the commercialized case of the lattice-matched Ga_0.49_In_0.51_P/Ga_0.99_In_0.01_As/Ge TJSCs, the bandgap energies are 1.9, 1.4, and 0.67 eV, respectively, which means each subcell has its very specific absorption spectrum [11,12,13]. Hence, the maximum number of photons absorbed by each subcell seems to be expected only if their optical thickness was thick enough. However, the short minority carrier lifetime of a few nanoseconds present in GaInP film limits the typical minority carrier diffusion lengths [14]. Consequently, the thickness of GaInP top-cell is generally allowed in the order of hundreds of nanometers for carrier collection other than its theoretical optical thickness of micrometers, resulting in unsatisfactory absorption of incident solar radiation [15]. Meanwhile, taking the material consumption into account, it also motivates a reduction in thickness of the active region of solar cells [16]. Therefore, a feasible and effective approach should be explored urgently for solving the puzzle between sufficient light absorption and efficient carrier collection in GaInP top-cell.

Most recently, an appealing approach involves the plasmonic nanostructures. The advantages of surface plasmon excitations is attributed to two aspects. The first involves an obviously increased material extinction for incident light arising from an enhanced local electromagnetic field near the nanostructures [17]. The other relates to the extended incident light path owing to a strong scattering of incident light into the active region of solar cells, with a result of increase in absorption and the promotion of photovoltaic conversion efficiency [18]. To date, many studies provide insights into the metal plasmonic nanostructures and, therefore, contribute greatly to practical photovoltaic applications [19,20,21,22,23]. However, few investigations are available on clarifying the feasibility of plasmonic nanostructures for GaInP/GaInAs/Ge TJSCs because the thermal treatment process for fabrication nanoparticles (NPs) is easy to cause the dopant diffusion in the highly-doped tunnel junction [24]. It is well known that annealing metal film is the simplest method to obtain metal nanoparticles. Unfortunately, too low an annealing temperature is not suitable for the formation of metal NPs, and too high an annealing temperature will greatly degrade the performance of TJSCs derived from the diffusion of dopant in TJSCs [25]. However, double hetero-structure tunnel junctions with wider bandgaps and lower diffusion coefficients are known for effectively suppressing impurity diffusion [13], so the problem of impurity diffusion in tunnel junctions is not an insurmountable barrier for improving the photocurrent of GaInP top-cells. Therefore, exploiting appropriate thermal treatment conditions for silver (Ag) NPs without inducing the dopant diffusion is an important strategy needing further study. Herein, the present work was aimed at promoting the photocurrent of GaInP top-cell using plasmonic nanostructures and, therefore, the efficiency of TJSCs.

In this work, Ag NPs were adopted because their extinction spectral range [26] is almost perfectly matched with the absorption range of the GaInP top-cell, and a series of experiments on annealing temperature, exposure time, and heating rate were proceeded to facilitate the light absorption of GaInP top-cell. We found that both the experimental and theoretical study confirmed the ellipsoidal Ag NPs annealed at 350 °C show a better extinction performance than the spherical Ag NPs annealed at 400 °C. The photovoltaic conversion efficiency of the device with ellipsoidal Ag NPs reaches 31.02%, with a nearly 5% relative improvement in comparison with the device without Ag NPs. The findings provide a feasible, cost-effective solution to solve the puzzle between the carrier collection and optical absorption in GaInP top-cells.

## 2. Experiments

**Figure 1:**
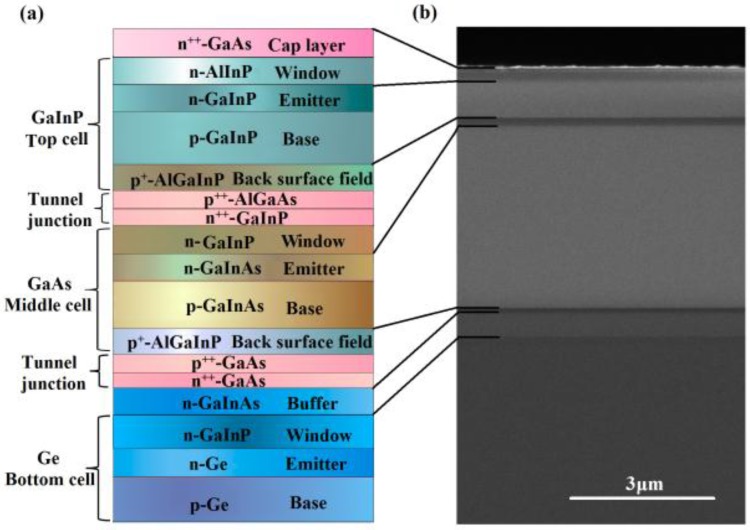
(**a**) Schematic layer structure and (**b**) cross-sectional view scanning electron microscope (SEM) image of the epitaxial structure of GaInP/GaInAs/Ge triple-junction solar cells (TJSCs).

The material growth of TJSCs on a p-type Ge substrate was performed by metal-organic vapor phase epitaxy (MOVPE) and the growth conditions were similar to those described elsewhere [27]. Figure 1a shows a schematic illustration of the material structure of the GaInP/GaInAs/Ge TJSCs evaluated in this work. The overall layer structure for the cell contains three pn-junctions and two tunnel junctions between the subcells. The Ga_0.51_In_0.49_P top-cell, Ga_0.99_In_0.01_As middle-cell, and Ge bottom-cell are all lattice-matched, which can effectively avoid the formation of dislocations and ensure excellent material quality. The three subcells were series connected with two highly-doped and ultra-thin tunnel junctions, which are favorable for a low resistance and high current density. The device processing on ohmic contacts, wet etching, and anti-reflection coatings were performed after the theoretical design of electrode patterns and anti-reflection coatings. The cell size was 10 × 11 mm^2^ and 1 mm grid pitch of electrode was designed for an optimal electrode distribution under one-sun operation. The performance characterization of current density-voltage (*J*-*V*) characteristic curves and external quantum efficiency (EQE) for devices without Ag NPs has been measured. The device parameters of short-circuit current density, open circuit voltage, and efficiency are averaged from 12 individual devices with spherical or ellipsoidal NPs, respectively.

**Figure 2:**
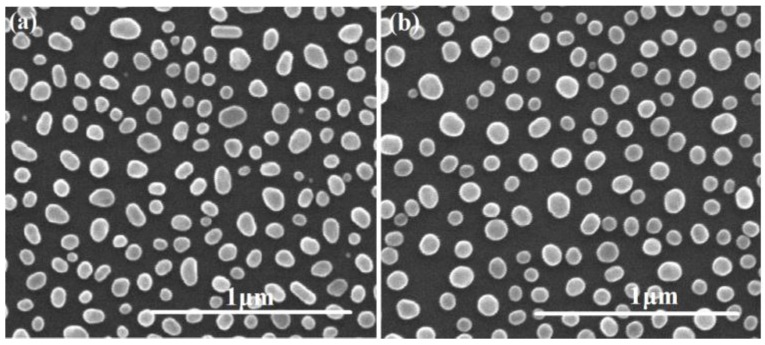
Plane-view SEM images of (**a**) ellipsoidal and (**b**) spherical Ag NPs.

Then, Ag films with 7 nm thickness were deposited on TJSCs at room temperature by magnetron sputtering, and the detailed fabrication processes can be referred to our previous work [18]. In consideration of the growth temperature for epitaxial layers is about 600–700 °C, the annealing temperature and temperature fluctuation for Ag film were strictly controlled. To the former, it is no higher than 450 °C, and to the latter, it is no more than 10 °C according to the computer supervising system. A series of experiments were performed for obtaining an optimal annealing temperature, exposure time, and heating rate. Lastly, the parameters were given as the following: the Ag films were annealed at 350 and 400 °C for 10 min under a nitrogen atmosphere with a heating rate of 150 °C/s. The resulting samples of ellipsoidal and spherical Ag NPs were shown in Figure 2.

Surface morphologies of Ag NPs were observed by a NOVA NANOSEM 650 scanning electron microscopy (SEM, FEI, Brno, Czech Republic). The optical properties were measured by an ultraviolet-visible spectrophotometer (UV spectrophotometer, Cary 5000, Varian, Palo Alto, CA, USA). The current density versus voltage (*J*-*V*) characteristic was tested by New-port 92250A Solar Simulator (Newport, Irvine, CA, USA) under one-sun AM1.5 (1000 W/m^2^, 25 °C) standard test conditions. The light response property of the devices was further investigated using Qtest-2000 external quantum efficiency (EQE) systems (Crowntech, PA, USA).

## 3. Results and Discussion

### 3.1. Epitaxial Structure and Performance of GaInP/GaInAs/Ge TJSCs

**Figure 3:**
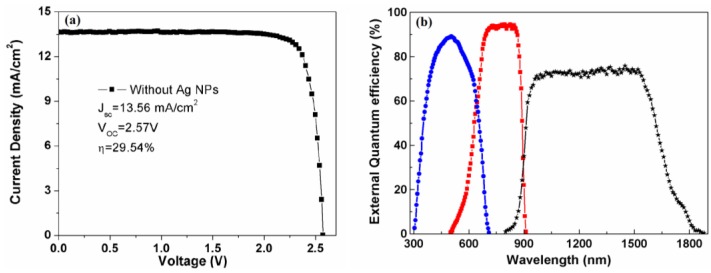
(**a**) Current-voltage (*J*-*V*) characteristic curve and (**b**) external quantum efficiency of the GaInP/GaInAs/Ge device.

Figure 1b demonstrates the cross-sectional view SEM image of the epitaxial structure of the GaInP/GaInAs/Ge TJSCs. As can be seen, the cell has a clear configuration and distinct interfaces, and the epitaxial structure mainly includes a 0.40-μm-thick GaInAs buffer layer, a 3.75-μm-thick middle-cell and a 0.75-μm-thick top-cell, as well as two 0.03-μm-thick tunnel junctions. The thickness of each layer, the doping concentration level and the material composition are based on the theoretical optimization. The *J*-*V* characteristic curve of TJSCs without Ag NPs under AM 1.5G solar irradiation (100 mW/cm^2^) is presented in Figure 3a. As can be seen, the short-circuit current density (*J*_sc_), open circuit voltage (*V*_oc_), and photovoltaic conversion efficiency (η) are 13.56 mA/cm^2^, 2.57 V and 29.54%, respectively.

Previous researches demonstrate that the *J*_sc_ of TJSCs is limited by one of the subcells [28,29], but the *J*_sc_ of each subcell cannot be directly obtained by *J*-*V* measurement. Therefore, the light response property of the devices was measured to calculate the *J*_sc_ of each subcell using EQE. Figure 3b exhibits the EQE of GaInP, GaInAs, and Ge subcells for the TJSCs without Ag NPs. Here, the absorption edges of GaInP, GaInAs, and Ge subcells are 670, 900, and 1800 nm, respectively. The EQE curves of the top- and middle-cell both drop rapidly near their absorption edges *versus* the gradual-decreasing modes of Ge bottom cell when wavelength ranges from 1600 to 1800 nm, which stems from the severe back surface recombination of Ge bottom-cell. The integrated current density for top-, middle- and bottom-cell is 13.70, 14.21, and 18.35 mA/cm^2^, respectively, which is in good agreement with the *J*_sc_ (13.56 mA/cm^2^) of the *J*-*V* measurement. It can be concluded that the *J*_sc_ of the TJSCs is limited by the GaInP top-cell, and an appropriate approach of increasing photocurrent of the top-cell is indispensable for high-performance TJSCs.

### 3.2. Morphology and Optical Properties of Ag NPs

Figure 2 presents the plane-view SEM images of the samples of Ag films annealed at 350 and 400 °C, respectively. As seen in Figure 2a, the Ag film was transformed into ellipsoid-like Ag NPs with smooth surface after annealing at 350 °C. The average principal axes length 2*a*–*c* of these ellipsoidal particles are 76, 94, and 76 nm. While the Ag NPs annealed at 400 °C appear very nearly spherical in shape with an average diameter *D* of 88 nm, as shown in Figure 2b. The average sizes of those ellipsoidal and spherical particles in Figure 2a,b are statistically determined by ImageJ software (National Institutes of Health, Bethesda, MD, USA). Hence, the Ag NPs exhibit obviously different morphology features at the two annealing temperatures of 350 and 400 °C, and the shape the Ag NPs tends to be ellipsoid-like at lower annealing temperatures.

**Figure 4:**
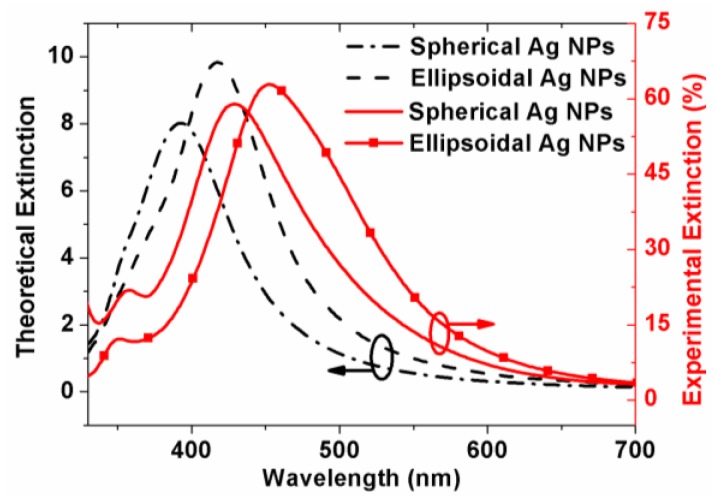
Theoretical (dotted lines) and experimental (solid lines) extinction spectra of spherical Ag NPs with *D* = 88 nm and ellipsoidal Ag NPs with 2*a*/2*b*/2*c* = 76/94/76 nm.

The insights into the metal plasmonic nanostructures require a deep understanding of their optical properties. Figure 4 displays both the experimental and theoretical extinction spectra for ellipsoidal and spherical Ag NPs. For comparison, the experimental results are marked with solid lines and the theoretical extinction spectra are marked with dashed lines. Samples of those Ag NPs fabricated on glass with similar process conditions were used to carry out the extinction spectra measurement. The theoretical extinction efficiency was calculated by the discrete dipole approximation (DDA) method [30]. In our calculation, both the perpendicular and parallel polarizations were assumed to have the same proportion of 50%, and the dielectric constant of Ag nanoparticles was extracted from [31].

As shown here, the theoretical calculation is comparatively consistent with the experimental result. The most significant aspect is that both the theoretical and measured extinction spectrum ranges are almost perfectly matched with the absorption wavelength range of GaInP top-cell [26], especially the measured one. The second aspect is that ellipsoidal Ag NPs demonstrate better extinction performance than that of spherical Ag NPs. Finally, the increased extinction ability almost disappears for wavelength beyond 600 nm.

Meanwhile, there are two different features exhibited in Figure 4. Firstly, the theoretical dipole extinction peaks are located at 393 and 418 nm for ellipsoidal and spherical Ag NPs, respectively, and the corresponding experimental dipole extinction peaks are located at 429 and 453 nm. Apparently, a red-shift of about 35-nm, both for the experimental extinction peak of ellipsoidal and spherical Ag NPs, appears, which can be attributed to the retardation effects occurred on the particles [32] and the practical wider size distribution. Secondly, the broadening of full width at half maximum (FWHM) of experimental results can be explicated by the wide size distribution of NPs. Overall, the features of the red-shift, the broader FWHM, and higher peak intensity are beneficial for harvesting more incident sunlight.

### 3.3. Enhanced Performance of GaInP/GaInAs/Ge TJSCs by Ag NPs

**Figure 5:**
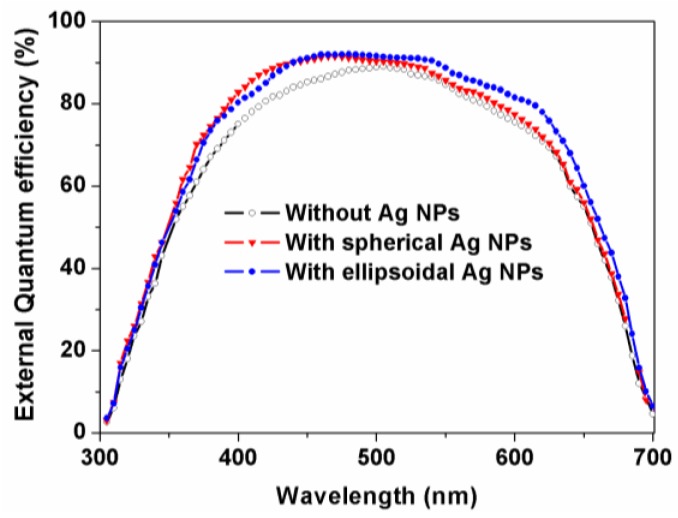
External quantum efficiency of GaInP top-cell in TJSCs.

Since the extinction spectra of Ag NPs cover from 300 to 600 nm, we speculate that the plasmonic nanostructures have little effect on the middle- and bottom-cell. To verify the impact of plasmonic nanostructures, the EQE of GaInP/GaInAs/Ge TJSCs with Ag NPs was measured. As expected, only the spectral response characteristic of GaInP top-cell is affected by Ag NPs. Hence, Figure 5 gives the EQE of GaInP top-cell with and without Ag NPs. As shown in Figure 5, the EQEs of devices with Ag NPs are higher than that of device without Ag NPs. The improved EQE for a device with spherical Ag NPs mainly locates from 390 to 490 nm, which is in accordance with the extinction spectra. Similarly, the excellent extinction performance of ellipsoidal Ag NPs leads to a higher EQE of the top-cell in a broader spectral range. The integrated current for devices without and with spherical and ellipsoidal Ag NPs is 13.70, 14.09, and 14.31 mA/cm^2^, respectively. Definitely, distinct photocurrent enhancements are obtained both for spherical and ellipsoidal Ag NPs compared with that of the device without Ag NPs, especially for the latter. The results allow the conclusion that ellipsoidal Ag NPs with better extinction property can obviously enhance the performance of TJSCs.

**Figure 6:**
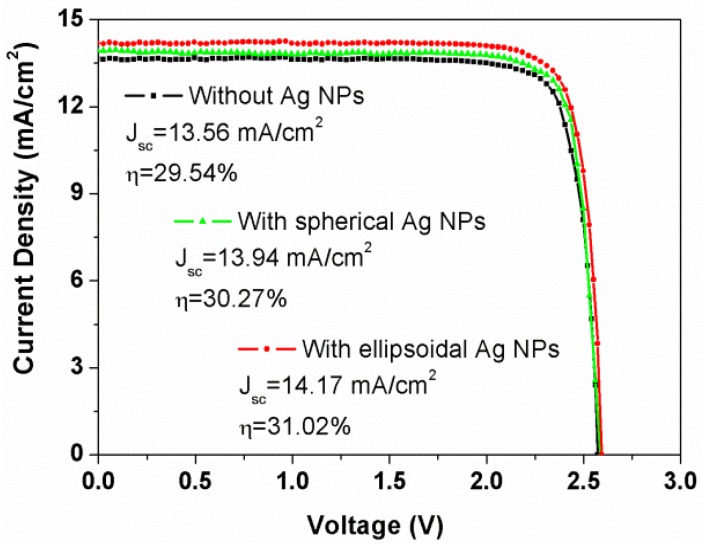
*J*-*V* characteristics of GaInP/GaInAs/Ge triple-junction solar cells at one-sun AM 1.5G, 25 °C. The measurement was performed on the 1.1 cm^2^ solar cell devices.

Figure 6 presents the *J*-*V* characteristic curves of TJSCs with and without Ag NPs under AM 1.5G solar irradiation. As demonstrated in Figure 6, there is no obvious difference in the *V*_oc_ for all devices, but their *J*_sc_ and η increase when Ag NPs are incorporated into the TJSCs. This could be attributed to the increased material extinction of Ag NPs within the spectral range from 300 to 600 nm. For the device without Ag NPs, the *J*_sc_ and η are 13.56 mA/cm^2^ and 29.54%, respectively. The η for devices with spherical and ellipsoidal Ag NPs is improved from 30.27% to 31.02%, respectively. This can be attributed to the key factor of *J*_sc_, which increases from 13.94 to 14.17 mA/cm^2^ and is in accordance with the EQE results discussed in the previous part. From the results, we conclude that Ag NPs, especially ellipsoidal Ag NPs, are obviously beneficial for the performance of TJSCs, but it is still a challenge to fabricate of metal NPs without inducing the dopant diffusion of tunnel junctions.

## 4. Conclusions

In conclusion, we demonstrated a simple approach for increasing the photocurrent of the top-cell using plasmonic nanostructures for current-matched GaInP/GaInAs/Ge TJSCs. The devices with ellipsoidal Ag NPs display an enhanced photocurrent due to the remarkably increased material extinction. Both the experimental and theoretical results verify the ellipsoidal Ag NPs with the lower annealing temperature of 350 °C show excellent optical properties. Under the illumination of AM 1.5G 100 mW·cm^−2^, the photovoltaic conversion efficiency of the device with ellipsoidal Ag NPs reaches 31.02%, with a nearly 5% relative improvement in comparison with the device without Ag NPs (29.54%). The findings of this study elucidate that plasmonic nanoparticles located on the illuminated surface of a solar cell is a promising structure for resolving the puzzle between carrier collection and optical absorption in GaInP top-cells and facilitating the light trapping of TJSCs.
